# Introducing the Algerian Mitochondrial DNA and Y-Chromosome Profiles into the North African Landscape

**DOI:** 10.1371/journal.pone.0056775

**Published:** 2013-02-19

**Authors:** Asmahan Bekada, Rosa Fregel, Vicente M. Cabrera, José M. Larruga, José Pestano, Soraya Benhamamouch, Ana M. González

**Affiliations:** 1 Department of Biotechnology, Faculty of Sciences, University of Oran, Oran, Algeria; 2 Department of Genetics, Faculty of Biology, University of La Laguna, La Laguna, Tenerife, Spain; 3 Department of Genetics, Faculty of Medicine, University of Las Palmas de Gran Canaria, Las Palmas de Gran Canaria, Gran Canaria, Spain; 4 Forensic Genetics Laboratory, Institute of Legal Medicine of Las Palmas, Las Palmas de Gran Canaria, Gran Canaria, Spain; IPATIMUP (Institute of Molecular Pathology and Immunology of the University of Porto), Portugal

## Abstract

North Africa is considered a distinct geographic and ethnic entity within Africa. Although modern humans originated in this Continent, studies of mitochondrial DNA (mtDNA) and Y-chromosome genealogical markers provide evidence that the North African gene pool has been shaped by the back-migration of several Eurasian lineages in Paleolithic and Neolithic times. More recent influences from sub-Saharan Africa and Mediterranean Europe are also evident. The presence of East-West and North-South haplogroup frequency gradients strongly reinforces the genetic complexity of this region. However, this genetic scenario is beset with a notable gap, which is the lack of consistent information for Algeria, the largest country in the Maghreb. To fill this gap, we analyzed a sample of 240 unrelated subjects from a northwest Algeria cosmopolitan population using mtDNA sequences and Y-chromosome biallelic polymorphisms, focusing on the fine dissection of haplogroups E and R, which are the most prevalent in North Africa and Europe respectively. The Eurasian component in Algeria reached 80% for mtDNA and 90% for Y-chromosome. However, within them, the North African genetic component for mtDNA (U6 and M1; 20%) is significantly smaller than the paternal (E-M81 and E-V65; 70%). The unexpected presence of the European-derived Y-chromosome lineages R-M412, R-S116, R-U152 and R-M529 in Algeria and the rest of the Maghreb could be the counterparts of the mtDNA H1, H3 and V subgroups, pointing to direct maritime contacts between the European and North African sides of the western Mediterranean. Female influx of sub-Saharan Africans into Algeria (20%) is also significantly greater than the male (10%). In spite of these sexual asymmetries, the Algerian uniparental profiles faithfully correlate between each other and with the geography.

## Introduction

On geographic, archaeological and historical grounds, Northwest Africa is considered a distinct spatial-temporal entity [1]. The core of this region comprises Morocco, Algeria and Tunisia, but sometimes also includes Libya and Mauritania. The region was known as Africa Minor by the ancients and as the Maghreb by the Arabs, the far western region of their Empire. From the Middle Paleolithic on, while the Neanderthals occupied Europe and Western Asia, anatomically modern humans with their Aterian industry already flourished in the Maghreb. After a prolonged hiatus but still in Paleolithic times, a new backed bladelet industry, named Iberomaurusian, replaced the Aterian in this area [2]. A wet period beginning around 9,000 years ago brought Saharan and Mediterranean Neolithic influences to the autochthonous Capsian Epipaleolithic. It seems that since that time Berber Afroasiatic dialects gave some cultural homogeneity to the anthropologically diverse populations of the Maghreb [3]. In historical times, North Africa was affected by the expansion of several Mediterranean civilizations, particularly the Phoenicians and Romans, who left their cultural influences with only minor demic impact on the Berber population [3]. However, the Berber language was not seriously threatened until the Islamic Arabs expanded their religion and culture to the Maghreb, since the end of the 7th century onwards [4]. Widely superseded by Arabic, Berber dialects are today confined to the more mountainous and desert rural areas of the region.

Population genetic studies, mainly those using the non-recombining Y-chromosome and mitochondrial DNA (mtDNA) uniparental polymorphisms, have provided insight into not only the structure and relationships among North African and neighboring populations but also the most probable origin and date of past immigrations and expansions of several informative lineages in the area. From the beginning, a prominent mtDNA Euroasiatic genetic component was observed in the Northern areas occupied by Morocco [5] and Egypt [6], with gradual sub-Saharan African influences moving southwards to the Western Sahara and Mauritania, or to Nubia and the Sudan respectively. Populations from the Sahel belt and the Chad basin almost certainly played an important role in this sub-Saharan-North African connection [7]–[9]. Comparisons between North African and Mediterranean Europe maternal and paternal gene pools [10]–[13] reveal sharp discontinuities and limited gene flow between both areas. Furthermore, Berbers constitute a very heterogeneous group showing significant differences even between geographically close communities [14]–[20]. However, an unexpected lack of differentiation between Berber and Arab speaking communities was found [15], [21]–[23].

These results suggest that the Arabization phenomenon was mainly an acculturation process of the indigenous Berber population. However, the significantly higher presence of the prominently Arab Y-chromosome J-M267 haplogroup in cosmopolitan compared to rural samples pointed to a substantial male-biased Arab influence in North Africa and the Levant [11], [15], [16], although it is probable that the diffusion of Islam only reinforced previous human displacements [24], [25]. Interestingly, wide geographical longitudinal gradients are detectable overlying local microstructure in North Africa for several uniparental markers [15], [17], [26], [27]. Some of these lineages, such as the mtDNA haplogroups U6 [28]–[30], M1 [29], [31], [32] and X1 [33] had their ancestral roots in the Middle East but expanded in North Africa since Paleolithic times with instances of secondary dispersion in this area. Others, like sub-haplogroup U5b1b [34], sub-haplogroups H1 and H3 [20], [35], [36] and haplogroup V [37] seem to have reached North Africa from Iberia in a post-last glacial maximum expansion. In concordance, an ancient DNA study from Ibero-Maurusian bone remains from Taforalt in Morocco detected the presence of haplogroups U6, V, T and probably H, pointing to a Paleolithic genetic continuity in Northwest Africa [38]. Additionally, male lineages also provide support to a Paleolithic Asia to Africa back migration [39] with Holocene trans-Saharan spreads as testified by the haplogroup R-V88 distribution [40]. Other lineages, E-M81 [26] and E- M78 [41], seem to be of North African origin with Paleolithic and Neolithic expansions that reached surrounding areas. The presence of these clades in southwestern Europe has been attributed to trans-Mediterranean contacts without involving the Levant [41], [42].

The impressive genetic information gathered from North Africa is beset with a notable gap, the lack of consistent information for the Algerian populations. Algeria is the largest country of the Maghreb and, in fact, the largest country of the whole continent. Although at mtDNA sequencing level the first North African sample studied was from an Algerian Berber-speaking Mozabite population [43], it resulted to be a very isolated group not representative of the whole Algerian population. After that, only a small sample of miscellaneous Algerians has been analyzed [13]. Similarly, only small samples of Algerian Arabs and Berbers have been studied with Y-chromosome binary polymorphisms [26]. To fill in this gap we analyzed a representative cosmopolitan sample from the Oran area of northwestern Algeria. We chose an urban area because urban populations give more representative information than rural, often isolated, localities [15]. In addition, Oran is considered the second largest city in Algeria and lies near Siga, one of the main cities of the largest Algerian Berber kingdoms in classical times [3]. In this study we characterized 240 maternally unrelated Algerians from this area by mtDNA HVS-1 region sequencing and haplogroup diagnostic coding positions by RFLP and SNaPshot multiplexing in order to obtain their maternal profiles. The male sub-set of this sample (102 paternally unrelated males) was previously analyzed for Y-chromosomal binary markers and short tandem repeat haplotypes [44]. However, in the present study, this male sample was further genotyped for the recently described informative Y-chromosome polymorphisms within haplogroups E [41] and R [45] whose subdivision has increased the phylogeographic differentiation between Europe and North Africa. Furthermore, in order to obtain more accurate comparisons, we extended these Y-chromosome fine resolution analyses of haplogroups E1b (M78) and R1b (M343) to published samples of Iberians and Moroccans [46], Saharawi and Mauritanians [47] and Tunisians [15]. This uniparental genetic information has been used to integrate Algeria into the overall North African genetic landscape.

## Materials and Methods

### Samples

Blood samples were anonymously obtained from a total of 240 unrelated healthy adult residents in northwest Algeria (Oran area) after informed written consent. This study was approved by the research ethics committee of the University of La Laguna. DNA was extracted using the standard salting-out method [48]. For Y-Chromosome analysis, DNA from previous surveys were used for fine resolution analyses of haplogroups E1b (M78): 26 samples from Iberians and Moroccans [46], 6 from Algerians [44], and 4 from Tunisians, and for R1b (M343): 361 samples from Iberians and Moroccans [46], 13 from Saharawi and Mauritanians [47], 12 from Algerians [44], and 2 from Tunisians.

### Mitochondrial DNA analysis

Mitochondrial DNA (mtDNA) sequence analysis was performed on the regulatory hypervariable segment I region (HVS-1). Haplogroup diagnostic mutations were analyzed using PCR-RFLPs and/or SNaPshot multiplex reactions [49], [50]. HVS-1 mtDNA segments were PCR amplified using primers pairs L15840/H16401 as previously described [51]. Successfully amplified products were sequenced for both complementary strands using the DYEnamic™ ET Dye Terminator Kit (Amersham Biosciences), and samples were run on MegaBACE™ 1000 (Amersham Biosciences) according to the manufacturer's protocol. To refine the haplogroup assignation made on the basis of HVS-1 sequences, 16 SNPs were analyzed on 93 chosen samples (See Supplementary Table S1).

Samples belonging to haplogroup H were assorted into H subgroups (H1–H15 subhaplogroups) using two SNaPshot multiplex reactions as described by Quintáns et al. [49] and Grignani et al. [50]. A selection of 12 SNPs defining common European haplogroups were analyzed by an additional SNaPshot multiplex reaction as described by Quintáns et al. [49]. Further SNP analyses were performed on three samples (ALG101, ALG196 and ALG205) by sequencing (Supplementary Table S1), to genotype SNPs 13934; 5999; 6047; 1811; 14070 and 14139, which are diagnostic positions for subhaplogroups U3a; U4′9; U4; U2′3′4′7′8′9; U1 and U3, respectively. Fragments comprising these positions were PCR amplified and sequenced as previously described [52].

### Haplotype classification

RFLP analyses and subhaplogroup H nomenclature were as in Loogväli et al. [53] and Roostalu et al. [54]. Classification into other sub-haplogroups was performed, whenever possible, using the updated (mtDNA tree Build 15; 30-9-2012) nomenclature proposed by van Oven and Kayser [55].

### Y-Chromosome analysis

**Figure 1:**
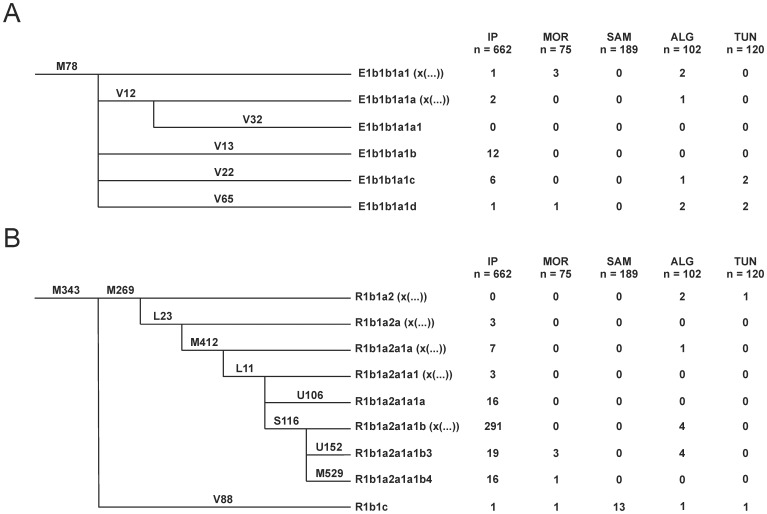
Simplified phylogenetic trees for Y-chromosome sub-haplogroups. A) E-M78 and B) M-R343.

For updated subdivision of haplogroup E we amplified and analyzed SNPs V12, V13, V22, V32 and V65 as specified by Cruciani et al. [41], [56]. For updated subdivision of haplogroup R, SNP V88 was amplified and analyzed as in Cruciani et al. [40] and SNPs L11, L23, M412, M529, S116, U106 and U152 as in Myres et al. [45]. Samples typed for these markers are detailed in Figure 1. Haplogroups were formally designated according to Karafet et al. [57], and refined following the International Society of Genetic Genealogy website (http://www.isogg.org/tree/). However, for markers subdividing E-M78 and R-M343 the nomenclature proposed by Cruciani et al. [41], [56] and Myres et al. [45], respectively, were used. To abbreviate, haplogroups were named only by their first letter and terminal marker throughout the text.

### Statistical analyses

In addition to our samples, a total of 18050 mtDNA HVS-1 sequences from the Mediterranean basin (12675), Middle East (4106) and Caucasus (1269) and a total of 19343 Y-chromosome samples from the same areas (11314, 4448, 3581, respectively) were included in the statistical analyses. To overcome microdifferentiation effects, samples were collapsed into larger areas or whole countries. In order to keep the highest SNP phylogenetic resolution for the Y-chromosome analysis, sub-haplogroup subdivisions in total samples were, sometimes, extrapolated from partial samples of the same area in which these sub-haplogroups were typed [58]. However, when this was not possible, frequencies from different markers were aggregated and named by the haplogroup most ancestral marker.

Haplogroup diversities (h) were calculated according to Nei [59]. For the mtDNA H haplogroup dissection analysis, only HVS-1 positions from 16024 to 16365 were used for genetic comparisons of partial sequences with other published data. Genetic variation for both uniparental markers was apportioned within and among geographic regions using AMOVA by means of Arlequin software version 3.5 [60]. Populations from the North and South sides of the Mediterranean Basin were considered in assessing the maternal and paternal genetic structure of the North West Algerian population. ‘North Africa’ included samples from Egypt, Libya, Tunisia, Morocco, Mauritania and Western Sahara, and ‘South Europe’ included Spain, Portugal, France, Corsica, Italy, Sicily, Sardinia, Balkans and Crete. For more extended geographic comparisons the following areas were taken into account: the Caucasus, the Arabian Peninsula (including Saudi Arabia, Kuwait, UAE, Yemen, Oman and Dubai), Levant (containing samples from Jordan, Palestine, Syria, and Lebanon), Turkey, and Iran, as detailed in Supplementary Tables S2 to S5 for mtDNA and S6 and S7 for Y-chromosome.

Pairwise F_ST_ distances between populations were calculated from mtDNA and Y-chromosome haplogroup frequencies, and their significance assessed by a nonparametric permutation test as implemented in the Arlequin program [60]. Principal component analyses (PCA) were obtained with IBM SPSS Statistic 19 version (SPSS Inc., Chicago, Illinois).

Phylogenetic relationships among mtDNA HVS-1 sequences of subhaplogroup M1 were established using the reduced median network algorithm [61].

## Results

### Algerian mtDNA profile

**Figure 2:**
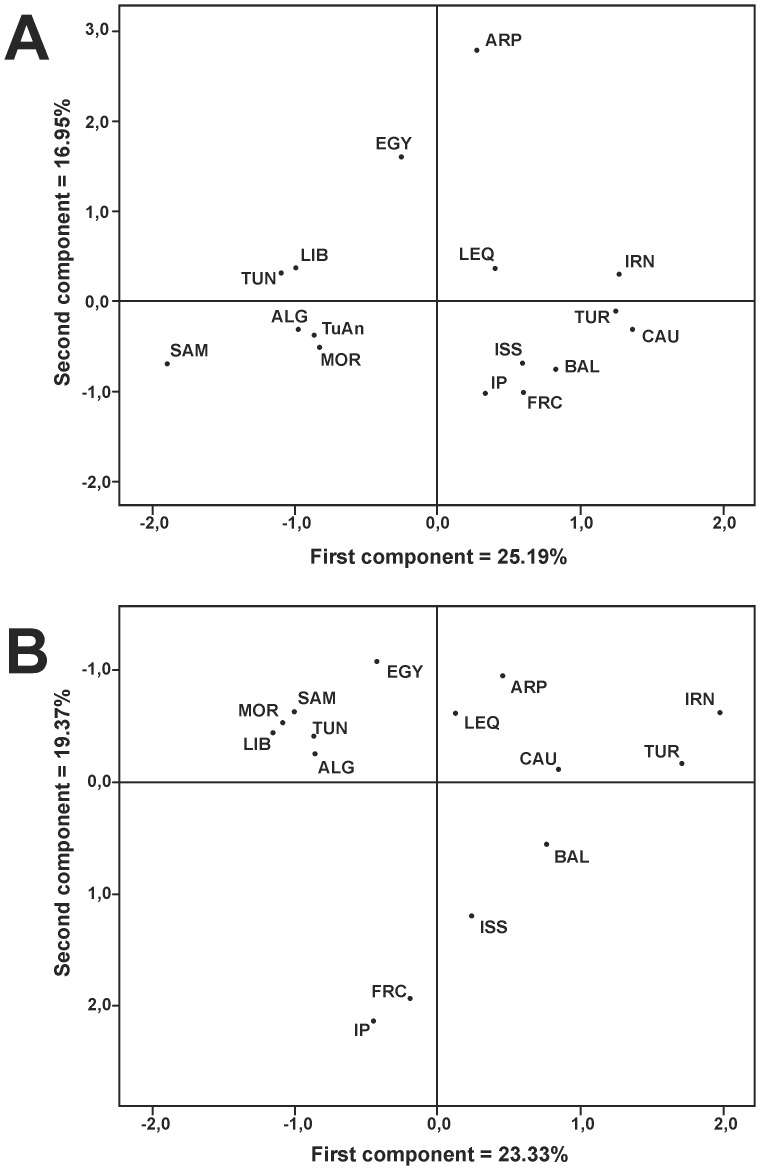
Graphical relationships among the studied populations. PCA plots based on mtDNA (a) and Y-chromosome (b) polymorphism. Codes are as in Supplementary Tables S2 and S6.

Pairwise comparisons between our Algerian sample and two ones published previously [13], [43] show that the three are heterogeneous in their haplogroup frequencies (Table 1). Mozabites are the most differentiated with a p<0.001 value in both comparisons, whereas the Oran-miscellaneous Algerian pair is only significantly different at p<0.05 level. A detailed inspection of their haplogroup profiles compared to those of surrounding populations (Supplementary Table S2) shows that Mozabites present a high excess of U3 and U6a1′2′3 haplotypes whereas the miscellaneous sample lacks HV0 representatives and has an outstanding excess of J/J1c/J2, L3e5 and L2a1 lineages. In contrast, Oran frequencies fall within the range expected by its geographic position, presenting only a slight deficit of K* lineages (p = 0.04) and a notable excess of M1 lineages (p = 0.002), which could be characteristic of Algeria since it is also shared by the miscellaneous sample (Supplementary Table S2). For these reasons we considered the Oran sample as the best representative of the general Algerian pool. However, in spite of their apparent differences, the three Algerian samples are joined as a tight cluster in a PCA analysis based on haplogroup frequencies (data not shown), and for this reason they have been pooled together for large-area comparisons. In addition, Andalusians from Tunisia show closer affinities with Moroccans and Algerians than with Tunisians (Figure 2A). In comparison with other Mediterranean and west Asian samples, the H haplogroup subdivision in the Algerian sample shows a typical Maghreb population structure (Supplementary Table S4). Congruently, the most common western subgroups, H1 (47.8%) and H3 (10.1%), represent 60% of H lineages. Furthermore, the H1 frequency in Algeria is intermediate between that found in Morocco (51.6%) and Tunisia (29.4%), fitting the eastward-decreasing gradient previously observed for this subgroup [36]. Thus, for the H haplogroup, Algerian affinities with the East seem to be weaker than with the West. Subgroups H2a1, H4 and H13a1 account for 42% of H lineages in Egypt but only 6% in Algeria (Supplementary Table S4). In addition, such a characteristic subgroup of the Arabian Peninsula as H6b (13%) was not found in our Algerian sample.

**Table 1:** mtDNA Haplogroup percentage frequencies in the Algerian populations.

	Populations		
Haplogroup	miscellaneous [13]	NW-Oran^1^	Mozabites [43]
**Sample size**	**47**	**240**	**85**
H/HV	29.79	30.83	22.35
HV1	2.13	-	-
HV0/HV0a/V	-	7.50	8.24
R0a	-	1.25	-
R*	-	0.83	1.18
U1a	-	0.83	-
U3b1a	-	1.25	10.59
U4	2.13	1.67	1.18
U5*	-	0.83	-
U5a	-	1.67	-
U5b5	2.13	-	-
U6a	-	2.50	1.18
U6a1′2′3	-	5.00	27.06
U6c	-	0.83	-
U8b/U2c	-	-	2.35
K*	4.26	1.67	-
T	-	1.67	-
T1a	2.13	3.33	4.71
T2	-	0.42	-
T2b*	2.13	2.50	-
T2c	-	0.83	-
T2h	-	0.42	-
J/J1c/J2	12.77	2.08	3.53
J1b2a	-	0.42	-
J2a2a1	-	0.42	-
J2b1	-	0.42	-
I	-	0.83	-
W	-	1.25	-
X	2.13	1.25	-
M1	12.77	7.08	4.71
N*/M*/L3*	-	0.42	-
L3b	2.13	-	-
L3b(16124!)	-	0.42	-
L3b1a3	-	0.83	3.53
L3b2a	-	0.42	-
L3d*	2.13	1.25	1.18
L3e1*	-	0.42	-
L3e2	-	0.83	2.35
L3e5	10.64	0.42	-
L3f*	-	0.83	-
L3f1b*	-	1.25	-
L4b2	-	0.42	-
L2*	-	0.83	-
L2a	-	1.25	-
L2a1*	6.38	0.83	-
L2a1a3	-	0.42	-
L2a1b	-	-	1.18
L2a1b′f	-	1.25	2.35
L2a1c2	-	0.42	-
L2a1(16189)	-	0.42	2.35
L2b1a	-	0.42	-
L2c1′2	-	2.08	-
L1b*	4.26	3.75	-
L1c*	-	0.83	-
L0f	-	0.42	-
L0a1	2.13	-	-

1 Present study.

Of all North African populations, Eurasian lineages are the most frequent in Algeria (80%) while sub-Saharan Africa origin accounts for the remaining 20%. At least two Eurasian lineages, M1 and U6, had Paleolithic implantation and subsequent expansions in North Africa, reaching the Sahel and Sudan belts. It seems that the main focus of distribution of U6 was in the Northwest and M1 in the Northeast areas of the Continent [28]–[31]. Indeed, the U6 haplogroup frequency is significantly higher in Algeria (11.83%) and W. Sahara and Mauritania (11.04%) compared to the eastern: Tunisia (5.24%, p<0.0001), Libya (4.08%, p = 0.006) and Egypt (0.77%, p<0.0001). However, the M1 frequency in Algeria (7.1%) raises an anomalous peak in its decreasing gradient from Northeast to Northwest (Supplementary Table S2). The rest of the Eurasian lineages in North Africa had a Levantine or Middle Eastern origin and, most probably, had reached Europe and Africa in parallel episodes in which sea-travel across the Mediterranean, occurring since Epipaleolithic times, played an important role [13], [62]–[66]. However, for some lineages present in North Africa but showing higher frequencies in Western Europe (for example, H1, H3, HV0 and U5b1b), a direct source in the Iberian Peninsula has been put forward, as a result of post glacial re-expansion [34], [35], [37], [53], [67]–[70]. The observed frequencies for H1 (47.8%), H3 (10.1%) and HV0 (7.5%) in our Algerian sample lie well within the Northwestern- to Northeastern- African decreasing gradients observed for these lineages, reaching statistical significance in the cases of H1 (r = −0.93; p = 0.008) and HV0 (r = −0.83; p = 0.043). Nevertheless, U5b1b haplotypes have not been found in Algeria yet, although this is a rare lineage in North Africa with its highest peak (6.2%) in the W. Saharan-Mauritanian region (Supplementary Table S2). Neither have we detected any representative of haplogroup U5b3, which expanded in Italy in Epipaleolithic times reaching nearby Mediterranean coasts [71]. However, a peculiar U5 haplotype (192 224 261 270) belonging to the U5b2b3 cluster [72] was observed. Until now, it has only exact matches with Hungarians [73], [74] and Romani from Bulgaria [75]. Regarding the sub-Saharan African component, Algeria (20%) is at the same level as Morocco (20.4%) and Egypt (22.9%) but significantly lower (p = 0.003) than Tunisia (30.1%) and marginally lower (p = 0.059) than Libya (27.1%). Aside from the widespread haplogroup L2a, the majority (14%) of Algerian L lineages (L1b, L2a1, L2b, L2c, L3b, L3d) are of West Africa origin. Those from Central Africa (L1c, L3e, L3f) account for an additional 5%, leaving around 1% for those of East African ancestry (L0, L3*, L4). It has been suggested that these lineages reached North Africa since Holocene times, when climatic amelioration permeated the Saharan desert. However the historical trans-Saharan slave trade promoted by the Arabs may have been mainly responsible for their present day incidence [9], [76].

The geographic origins and gradients of some of these haplogroups are graphically reflected in the PCA plot (Figure 2A). The first component clearly separates European Mediterranean populations from North African. Haplogroups N2, U2, T2 and I further separate the West Asian samples from European. On the other side, the sub-Saharan African haplogroups L3d, L1b, L2a1b′f, L2a1c1′2, L3b and U6a pull the North African countries to the left, leaving W. Saharan-Mauritanian as the most displaced. The second component aligns Mediterranean countries according to their geographic longitudinal transect, which continues through West Asia, leaving the Levant in an intermediate position. Haplogroups H, HV0, T2b, and to a lesser degree J2b1 and several U5 subgroups push the populations down whereas East African haplogroups such as L3i, L3x and L2a2 and eastern West-Eurasian haplogroups like N1, R0a and U9 pull them up.

Pairwise based F_ST_ distances between populations were calculated (Supplementary Table S8). Significant mean F_ST_ values were found between our Algerian sample and North African (0.023±0.002), European (0.036±0.003) and Middle East (0.021±0.003) populations. Within North Africa, the Algerian lowest genetic distance is observed for Tunisia (F_ST_ = 0.016) and the greatest for Egypt (0.026). Italy (F_ST_ = 0.032) and the Balkans (F_ST_ = 0.032) are the closest areas within the European peninsulas while France is the most distant European region (F_ST_ = 0.042). Finally, for the Middle East, the Levant (F_ST_ = 0.014) seems to be the most similar and the Arabian Peninsula (F_ST_ = 0.033) the most different. In fact, removing the pairwise comparison between Algeria and Arabia, the mean F_ST_ value for the Middle East drops considerably (F_ST_ = 0.018+0.001) rising the mean distance of Algeria from Europe significantly compared to that from the Middle East (p<0.01).

### Algerian Y-chromosome profile

Results for the sub-typing of haplogroups E-M78 and R-M343 in the Iberian Peninsula and Northwest African countries including Algeria are presented in Figure 1. In general, data for E-M78 agree with the previous analysis [41]. Therefore, the Eurasian E-V13 is the most common sub-group in Iberia, although one North African E-V65 type has also been detected. On the African side, the lack of E-M78 representatives in a total sample of 189 males from the W. Saharan-Mauritanian area is notable. For the Maghreb countries, the fact that the number of males belonging to para-group E-M78* is the same as those included in the autochthonous E-V65 group also stands out.

For the R-M343 subdivision, the Iberian Peninsula reflects a genuine European profile [45] except for the presence of one Sahel R-V88 type. In contrast, all R-M343 detected in W. Saharan-Mauritanian belong to sub-group R-V88, reaching a frequency of 7%, similar to those observed in other Sahel samples [40]. In the Maghreb countries, the frequency of R-V88 drops to around 1%. On the other hand, the presence in this area of representatives of the European sub-groups R-M412, R-S116, R-U152 and R-M529 points to North-South maritime contacts across the Mediterranean.

Supplementary Table S6 presents frequencies of Y-chromosome haplogroups, as spread out as possible, for the same countries-areas as performed for the mtDNA analysis. Clearly, markers E-V65, E-M81 and J1-M267 confirm the geographic and ethnic identity of Algeria but, while E-M81 represents an autochthonous group that sharply decreases in Egypt, J1-M267 points to a Levantine influence. Haplogroups G-M201, L-M20, R2-M124, T-M70, J2-M172 and the majority of derived J2 sub-groups all reflect West Asian influences on Europe with only weak inputs on North Africa. On their part, several European I sub-groups also extend to West Asia with minor gene flow to the African countries. Exceptions to this general pattern are the subgroups J2-M67 and R-M412 that have similar frequencies in Algeria as in Europe, and R2-M124 whose frequency in Egypt is not significantly different from the mean value of European and West Asian areas. These geographic influences are graphically reflected in the PCA analysis (Figure 2B). All the European countries are aligned in a diagonal transect running from the Iberian Peninsula to Turkey and the Caucasus, according to their respective geographic positions, and well separated from the North African countries. Within North Africa, the Maghreb region appears well differentiated from Egypt, which, reflecting its geographical position, is near to the Levant and the Arabian Peninsula. The most influential haplogroups in the first component separation are: E-M81, E-V65 and R-V88 that pull the North African countries together, and J-M172, R-M173, R-M17, R-M124 and R-L23 that pull West Asian countries in the opposite direction. In the second component, haplogroups R-L11, R-M529, R-U198, I-M223 and I-M26 are responsible for the spread of the European Mediterranean countries away from Egypt and Arabia, which in turn are pulled by J-M267, B-M60, E-V22 and E-M123.

Pairwise based F_ST_ distances between populations were calculated (Supplementary Table S8). The mean F_ST_ values comparing the Algerians with the other North African samples (0.061+0.015), with Europeans (0.197+0.019) and with West Asians (0.159+0.011) also reflects its geographic position. Within North Africa, its lowest distance is to W. Sahara-Mauritania (F_ST_ = 0.023) and the greatest to Libya (F_ST_ = 0.108). Italy (F_ST_ = 0.155) is the closest of the European peninsulas and Iberia the most distant (F_ST_ = 0.247), while for West Asia, the Levant (F_ST_ = 0.128) is the most similar area to Algeria and the Caucasus the most different (F_ST_ = 0.194).

Based on genome-wide genetic analysis, up to five differentiated genetic components (Maghreb, Near East, Europe, and west and east sub-Saharan Africa) were recently detected in the ancestry of North African populations [77]. Based on phylogeographic and relative gene diversity levels [78], we also tentatively decomposed Y-chromosome (Supplementary Table S9) and mtDNA (Supplementary Table S9) polymorphisms into the same five components (Table 2).

**Table 2:** Geographic components (%) considered in Y-chromosome and mtDNA lineages.

		Populations^1^															
	component^2^^,^ 3	IP	FRC	ISS	BAL	SAM	MOR	ALG	TUN	TuAn	LIB	EGY	CAU	TUR	LEQ	IRN	ARP
**mtDNA**	**EU**	56.7	45.9	46.2	24.2	29.8	34.9	29.9	24.6	13.5	28.6	7.9	17.9	11.1	10.2	10.0	3.5
	**ME**	38.1	52.2	51.2	74.5	17.1	31.7	31.4	36.9	21.3	36.8	58.1	80.6	84.0	76.3	85.1	71.4
	**NA**	2.0	0.3	1.3	0.4	11.7	14.7	20.7	10.8	7.7	7.1	9.9	0.4	0.0	2.3	0.4	3.2
	**EA**	0.8	1.0	0.4	0.8	2.1	3.2	1.9	8.0	4.5	6.3	12.4	0.9	3.8	4.1	4.0	12.6
	**WA**	2.4	0.6	1.0	0.3	39.3	15.5	16.1	19.8	18.7	21.2	11.6	0.1	1.0	7.2	0.4	9.1
**chrom Y**	**EU**	75.3	79.0	55.4	58.8	0.0	3.9	10.3	1.7	-	2.4	3.5	21.5	19.9	19.5	13.3	5.2
	**ME**	16.2	18.7	36.3	39.1	13.8	9.4	29.5	23.5	-	2.4	46.2	77.7	72.3	58.5	80.0	76.4
	**NA**	5.8	0.8	4.4	0.9	55.6	73.9	50.0	68.9	-	50.6	33.0	0.4	1.3	9.6	3.5	3.1
	**EA**	1.8	1.4	3.1	1.2	11.6	5.8	1.9	3.0	-	0.0	11.4	0.5	5.9	6.0	1.4	9.4
	**WA**	0.9	0.1	0.8	0.0	19.1	7.0	8.3	3.0	-	44.6	5.9	0.0	0.6	6.4	1.8	6.0

1 IP = Iberian Peninsula; FRC = France+Corsica; ISS = Italy+Sardinia+Sicily; BAL = Balkans+Creta; SAM = Sahara+Mauritania; MOR = Morocco; ALG = Algeria; TUN = Tunisia; TuAn = Tunisian Andalusians; LIB = Libya; EGY = Egypt; CAU = Caucasus; TUR = Turkey; LEQ = Levant (Jordan; Syria; Palestine; Lebanon; Druze)+Iraq; IRN+Kurds = Iran; ARP = Arabian Peninsula (Saudi Arabia; Oman; Yemen; United Arab Emirates; Qatar; Dubai; Kuwait).

2 EA = East Africa; EU = Europe; ME = Middle East; NA = North Africa; WA = West Africa.

3 Haplogroup assigned to each geographic component are detailed in Supplementary Table S9.

## Discussion

**Figure 3:**
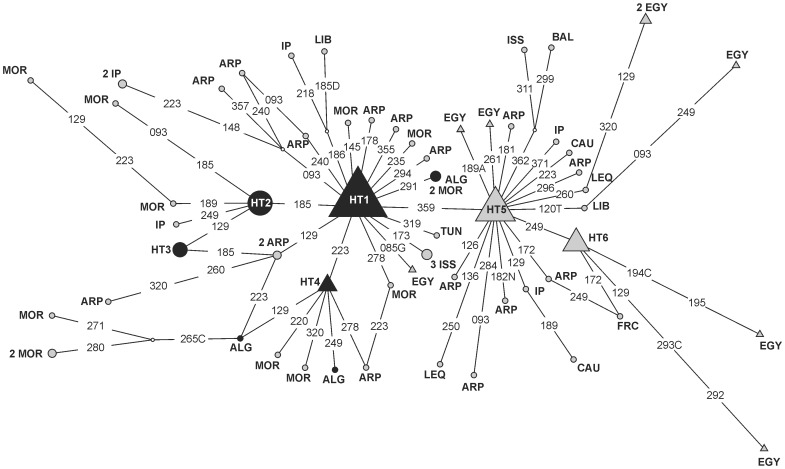
Reduced median network relating HVS-1 sequences of subhaplogroup M1. The central motif (haplotype HT1) differs from rCRS [90], [91] at position 16129 16189 16223 16249 16311. Population codes as in Supplementary Table S2. Numbers along links refer to nucleotide positions minus 16000; suffix indicates a transversion. Black circles correspond to haplotypes observed in Algeria, whereas grey triangles pentagons correspond to lineages found in Egypt. Haplotype observed both in Algeria and Egypt are indicated using a black triangle. Grey circles indicate haplotypes observed in other geographical regions. Size of boxes is proportional to the number of individuals included. HT1 = 13 ALG, 17 ARP, 2 BAL, 8 EGY, 5 IP, 5 ISS, 2 LIB, 18 MOR, 13 TUN, TuAn; HT2 = 6 ALG, ARP, BAL, IRN, LEQ, 2 LIB, 3 MOR, SAM, 2 TUN; HT3 = 2 ALG, ARP, LEQ, 2 MOR; HT4 = 3 ALG, 2 EGY, 3 IP, 2MOR; HT5 = 14 ARP, 3 BAL, 3 CAU, 10 EGY, 4 IP, IRN, 7 ISS, 4 LEQ, 2 LIB, 6 MOR; HT6 = ARP, 16 EGY.

Strong microdifferentiation has been detected for both uniparental markers in Tunisia [15]. It seems to be also the case for the Algerian mtDNA pattern as the three samples compared here showed significant differences among them. However, in spite of these differences, samples from the same country usually cluster together. A notable exception, for mtDNA, is the case of Andalusian from Tunisia that clustered with Moroccan samples. The presence in Algeria of a rare U5b2b3 haplotype, of Eastern Europe adscription, could be explained as result of the Ottoman influence. Although Algeria and W. Sahara-Mauritania show the highest frequencies for mtDNA haplogroup U6 in the Maghreb, division into subgroups reveals that whereas the majority of U6 haplotypes in W. Sahara-Mauritania (7.6%) are included in the ancestral cluster U6a, the bulk in Algeria (9.4%) belongs to derived subgroups U6a1′2′3 (Supplementary Table S2). Similarly, although Algeria (7.3%) and Egypt (8.5%) present the highest frequencies of the North African haplogroup M1, subdivision of this cluster shows clear phylogeographic differences; whereas 6.4% of the Egyptian lineages belong to the East African cluster M1a1, none M1a1 was found in the Algerian sample (Figure 3). These patterns are congruent with the existence of different origin of geographic spread for both haplogroups in the Maghreb and East Africa [28], [31]. Contrastingly, for the Y-chromosome North African autochthonous lineages E-V65 and E-M81, Algeria shows the lowest frequencies of all Maghreb countries (Supplementary Table S6). However, E-M81 frequencies in Algeria (44.2%) are still significantly higher (p<0.0001) than in Egypt (11.9%). These results confirm that for both uniparental markers, Egypt and to a lesser extent Libya stand out sharply from the Maghreb [16], [27].

Y-chromosome haplogroup J-M267 frequency is also the highest in Algeria. The presence of this clear Middle Eastern haplogroup in other areas has been attributed to prehistoric spreads [25], [79] and to the historic Islamic rule [15]. The localized distribution of the two most common haplotypes found in Algerians belonging to J1-M267 [44] points to an ancient implantation of this cluster in the country. However, the notable incidence of J2-M67 is most probably due to Aegean contacts [79], [80].

The unexpected presence of the European male lineages R-M412, R-S116, R-U152 and R-M529 in the Mahgreb could be the male counterpart of the maternal gene flow signaled by the mtDNA haplogroups H1, H3 and HV0. In fact, there are several haplogroups with clear geographical origins from European or North African sides of the Mediterranean, but also present on the opposite side. This could be used to estimate the respective levels of gene flow between areas, assuming that their present day frequencies in the source countries were the same when they spread to the other Mediterranean shore. Thus, mean frequency values for the native North African male clusters E-M81 and E-V65 in the Maghreb (Morocco, Algeria, Tunisia, Libya), are 40.03±11.66 and 3.40±0.60 respectively. The mean values for the same markers in western-central Mediterranean Europe (Iberian Peninsula, France and Corsica, Italy, Sardinia and Sicily) are 1.86±1.28 and 0.26±0.8 respectively. Taken together, these values would suggest around 5% male Maghreb input in Mediterranean Europe. In turn, E-V13, R-M412, R-S116, and R-U152 could be used to infer the male European input in the Maghreb, giving a value around 8%. Applying the same reasoning, mtDNA U6 and M1 frequencies on the European side would indicate the maternal gene flow from the Maghreb, the estimated value being around 10%. However, when we tried to calculate the European maternal input into the Maghreb using the H1, H3 and HV0 haplogroups, we realized that their respective mean frequencies in Mediterranean Europe (38.33+4.31, 17.27+3.57 and 5.23+1.06) are within the same range as those found in the Maghreb (42.05+4.92, 13.1+3.51 and 6.99+0.90). This would imply a 100% European contribution to the maternal pool of the Maghreb. The fact that the three markers show similar frequencies on both sides rules out stochastic processes as a possible explanation, but further analyses, based on complete mtDNA sequences, are mandatory to investigate alternative scenarios.

Genetic and geographic distances faithfully correlate for both uniparental markers (Figure 2), indicating populations from both sides of the Mediterranean remained apart until meeting in the Levant. This similarity allowed us to confront the main maternal and paternal discriminating contributors to the PCAs spatial distribution. Some equivalences are expected such as mtDNA U6a and Y-chromosome E-M81 and E-V65 affecting Maghreb countries, and that the West African mtDNA L clades and Y-chromosome R-V88 pulls W. Saharan-Mauritanian further over, or that the Mediterranean Europe distribution is largely determined by mtDNA and Y-chromosome lineages with origins and/or dispersions within Europe. However, these coincidences only reflect present-day frequencies, not common past histories. Furthermore, in spite of the similarities, differences among male populations are significantly greater than among the female. For instance, the mean F_ST_ distance between Algeria and other Maghreb countries for Y-chromosome (0.061) is nearly three times higher than for mtDNA (0.023), 5 times higher when based on distances between Algeria and Europe and nearly 8 times higher when involving Middle East populations. Gender specific demographic features were used to explain these differences [15]. There are also differences in male and female affinities between populations. Thus, Tunisia is the most related to Algeria at mtDNA level but W. Sahara-Mauritania is the closest when using Y-chromosome. Moreover, France is the most distant population from Algeria based on mtDNA but Iberia is the furthest when based on Y-chromosome. Finally, in the Middle East, Saudi Arabia is the less related population when comparing maternal profiles, but from the paternal view, the most distant area is the Caucasus. There are also coincidences; Italy is the closest European country to Algeria using both uniparental markers. Again, similarities and differences are apparent between both uniparental markers when differentiated genetic components of Maghreb, Near East, Europe, and west and east sub-Saharan Africa are taking into account. For the sub-Saharan East African component, Arabia and Egypt harbor the highest frequencies for both Y-chromosome and mtDNA. However, in the Maghreb, W. Sahara-Mauritania accumulates the maximum male eastern contribution and Tunisia the female one. Comparing the sub-Saharan West African component, the correspondence between male and female inputs is perfect; Iberia and Italy show the highest influences in Europe, W. Sahara-Mauritania and Libya in North Africa and the Levant and Arabia in the Middle East. For the European component, Iberia, France and Italy have the greatest representation in both uniparental markers, and for the Middle East it is the Caucasus. Nevertheless, in the Maghreb, the European mtDNA contribution in Morocco is the largest whereas Y-chromosome influence peaks in Algeria. Finally, the Middle East component shows congruent values for both markers, the Balkans is the region with the greatest Middle East component in Europe; Egypt has the greatest in North Africa and Iran in the Middle East. A big study concerning Y-chromosome in Iran has been published after this analysis was carried out [81], however haplogroup frequencies for both sets of Iranian samples are rather similar, and we do not think its inclusion would modify largely our conclusions.

Recently, it has been reported that the sub-Saharan African gene flow to Tunisia shows a strong sex bias, involving a significantly larger female contribution (p<0.0001) [15]. The same tendency holds for all North African populations except Libya, which could be attributed to insufficient sampling [19]. However, significance levels are more moderate in all instances; for example, probability values in Algeria (0.025) or in W. Sahara-Mauritania (0.043) are two times lower than for Tunisia. The same sex bias is found in the Middle East, reaching significance in Arabia (p = 0.0005) and in the Caucasus (p = 0.045). In Europe, only Italy shows significant differences (p<0.0001) for the gender contribution of sub-Saharan Africans but contrarily, in this case, the male input (3.91%) is highest than the female one (1.35%). On the basis of uniparental markers [82]–[84] and massive genomic analysis [77], [85], the bulk of the sub-Saharan African gene flow has been attributed to historic events such as Romanization, Islamic role and, even more so, the Arab and Atlantic slave trades. A preference for assimilation of females from minority ethnics groups in patriarchal societies has also been put forward [15], [82] to explain the general pattern of sub-Saharan African female integration. The case of Italy could be better explained, at least partially, by more ancient sub-Saharan African inputs into Europe than are thought by several authors to have occurred [83], [84], [86]. However, see Capelli et al. [87] for another point of view. All these uniparental peculiarities could be explained supposing: 1, the existence of several dispersion foci at different times in western Asia, independently influencing the African and European Mediterranean areas; 2, the spread of independent autochthonous lineages in both areas, and 3, bidirectional maritime contacts between areas with minor gene flow.

The inclusion of Algeria offers a more complete view of the North African genetic landscape from maternal and paternal perspectives, showing not only spatial gradients for some lineages but also sexual asymmetry in the relative affinities between populations. Perhaps, the strongest sexual discrepancy refers to the time of the main Middle East and European spreads into North Africa, whereas from the Y-chromosome perspective they seem to have occurred since the Neolithic onwards [26], [40], although see also Luis et al. [88]. From mtDNA [28]–[31] and wide genome analysis [77] the signals of Paleolithic influences are however evident. As the time to the most recent common ancestor through mtDNA is higher than that of the Y-chromosome [89], sex-specific demographic processes are probably the main factor behind this difference. A view reconciling the two perspectives would be that male lineages are better suited for detecting more recent human expansions whereas the ramifications of mtDNA genealogies extend to Paleolithic times and beyond.

## Supporting Information

Table S1 **HVS-1 sequences, SNPs and RFLPs analysis results for the North-west Algerian population (N = 240).**
(XLS)

Table S2 **mtDNA Haplogroup frequencies (%) in the studied populations.**
(XLS)

Table S3 **References of the populations studied in Table S2.**
(XLS)

Table S4 **Distribution of H subhaplogroup frequencies (%) in the studied populations.**
(XLS)

Table S5 **References of the populations studied in Table S4.**
(XLS)

Table S6 **Y-chromosome haplogroup frequencies (%) in the studied populations.**
(XLS)

Table S7 **References of the populations studied in Table S6.**
(XLS)

Table S8 **Pairwise linearized F_ST_ for mtDNA (above diagonal) and Y-chromosome (below diagonal) haplogroups.**
(XLS)

Table S9 **MtDNA and Y-chromosome geographic haplogroup assignation.**
(XLS)
